# Keap1 expression has independent prognostic value in pancreatic adenocarcinomas

**DOI:** 10.1186/s13000-015-0258-4

**Published:** 2015-04-16

**Authors:** Joel Isohookana, Kirsi-Maria Haapasaari, Ylermi Soini, Peeter Karihtala

**Affiliations:** Department of Pathology, Medical Research Center Oulu, Oulu University Hospital and University of Oulu, P.O. Box 50, 90020 Oulu University Hospital, Finland; Department of Pathology and Forensic Medicine, University of Eastern Finland, Cancer Center of Eastern Finland, PO Box 1627, 70211 Kuopio, Finland; Department of Oncology and Radiotherapy, Medical Research Center Oulu, Oulu University Hospital and University of Oulu, P.O. Box 22, 90029 Oulu University Hospital, Finland

**Keywords:** 8-hydroxydeoxyguanosine, Antioxidant enzymes, Keap1, Nrf2, Oxidative stress, Pancreatic cancer

## Abstract

**Background:**

Oxidative stress and redox-regulating enzymes may potentially accelerate pancreatic carcinogenesis and also affect chemoresistance. Recently major antioxidant response regulator NF-E2-related factor 2 (Nrf2) has been linked to poor prognosis in pancreatic cancer. Nrf2 activity is strictly regulated by oxidative stress sensor Kelch-like ECH-associated protein 1 (Keap1). Oxidative DNA damage can be estimated e.g. by 8-hydroxy-2′-deoxyguanosine (8-OHdG) expression. The aim of this study was to evaluate the expression and possible prognostic role of Keap1 and 8-OHdG in pancreatic cancer.

**Methods:**

We assessed immunohistochemically the expression of 8-OHdG and Keap1 in precisely characterized material of 69 pancreatic adenocarcinoma patients.

**Results:**

Nuclear 8-OHdG associated with cytoplasmic Keap1 expression (p = 0.031) and was overexpressed in patients with smaller tumors (p = 0.016) and in tumors without lymph node involvement (p = 0.051). Cytoplasmic 8-OHdG expression associated with higher differentiation (p = 0.023). Cytoplasmic Keap1 immunostaining associated with N0-staging (p = 0.0009) and the absence of distant metastases (p = 0.018). Membranous Keap1 associated with longer relapse-free survival (p = 0.041) and pancreatic cancer-specific survival (median survival 14 *vs*. 32 months; p = 0.029) and was in multivariate analysis an independent prognostic factor of pancreatic cancer-related death (HR 2.66, 95%CI 1.23-5.75).

**Conclusions:**

Oxidative stress and main redox regulators may participate in pancreatic carcinogenesis and Keap1 appears as a promising prognostic factor in pancreatic cancer. Future studies should also concentrate on potential link between redox regulation and chemoresistance in pancreatic cancer.

**Virtual slides:**

The virtual slide(s) for this article can be found here: http://www.diagnosticpathology.diagnomx.eu/vs/4220521801406476

## Background

Although pancreatic adenocarcinoma is not the one of the most common cancers, it has dismal prognosis, the five year survival being about 5% [1]. At the moment of diagnosis, malignant tissue extends beyond the pancreas in 90% of the cases and metastasis can often be detected [2]. Because of local expansion and early appearance of metastases, tumor is local enough for curative surgical resection only in 10-20% of the cases. Adjuvant chemotherapy with gemcitabine is considered in these cases, with slight improvement in survival [3].

Reactive oxygen species (ROS) are produced mostly as a consequence of aerobic metabolism in mitochondrial respiratory chain. In addition to their crucial role in signal transduction, ROS production is a cornerstone of radiotherapy and may also participate to gemcitabine resistance via hypoxia-inducible factor 1α- and nuclear factor κB- mediated CXCR4 up-regulation [4]. An expanding amount of literature from *in vitro* experiments suggests that ROS have a substantial effect on pancreatic carcinogenesis [5-9]. However, compared to most other solid tumors, these data from *in vivo* studies are rather limited in pancreatic cancer.

By definition, ROS have very limited lifetime, but their interaction with e.g. DNA can be reliably measured with the use of antibodies against specific footprints of oxidative damage. Hydroxyl radical (^•^OH) is the most reactive ROS with a lifetime of less than 1 nanoseconds and it reacts easily with pyrimidines, purines and chromatin protein resulting in base modifications, genomic instability and alterations in gene expression such as p53 tumor suppressor gene [10,11]. Nucleoside 8-hydroxy-2′-deoxyguanosine (8-OHdG) is a specific end-product of ^•^OH -derived damage in C8 carbonyl group of guanine and it is the most studied marker against oxidative damage in DNA [12].

Keap1/Nrf2 pathway is the key sensor of intracellular redox status. Under normal redox state, Keap1 (Kelch-like ECH-associated protein 1) is bound to Nrf2 (nuclear factor erythoid-derived 2-like 2) and Nrf2 activity is repressed by degradation in ubiquitin proteasome pathway [13-16]. When exposed to oxidative stress, Keap1 is inactivated and unable to bind Nrf2, which subsequently translocates to nucleus and binds to antioxidant response element (ARE) in DNA together with small Maf proteins [13,15]. This results in induction of several cytoprotective genes, including antioxidant enzymes, drug transporters and drug-metabolizing enzymes [13,15]. The importance of Keap1 is underlined in Keap1-deficient mice, which die postnatally and have a constitutive Nrf2 activation [17]. *In vitro* suppression of Keap1 in human prostate and non-small cell lung carcinoma cell lines results in an increased Nrf2 activity and further to sensitization to various chemotherapeutic agents and radiotherapy [18,19].

We recently demonstrated that nuclear Nrf2 expression associates with poor survival in pancreatic adenocarcinomas [20]. Partly as a continuation to that study, our specific aim was here to evaluate the role of both Keap1 and 8-OHdG in an independent material of pancreatic adenocarcinomas.

## Methods

### Samples

The study material consisted of 69 formalin-fixed, histological paraffin-embedded pancreatic adenocarcinoma samples. The samples were fixed in neutral formalin, embedded in paraffin blocks and stored at the Department of Pathology, Oulu University Hospital. Diagnoses were made during years 1993–2011 and 61 (88.4%) of them were diagnosed in 2000’s. All patients were diagnosed and treated at Oulu University Hospital, Finland. Only patients with at least palliative surgery were included to enable sufficient histological samples for reliable immunostaining evaluation. Mean follow-up time was 24.3 months (range 1–172 months). Patient characteristics are more precisely described in Table 1. The study was approved by the Ethics Committee of the Northern Ostrobothnia Hospital District and Finnish National Supervisory Authority for Welfare and Health.

**Table 1:** **Patient characteristics**

	**n (%)**
Tumor size (T)	
1	4 (5.8%)
2	23 (33.3%)
3	32 (46.4%)
4	7 (10.1%)
Missing	*3 (4.3%)*
Nodal metastasis (N)	
No	*34 (49.3%)*
Yes	*33 (47.8%)*
Missing	*2 (2.9%)*
Distant mestastasis (M)	
No	*59 (85.5%)*
Yes	*8 (11.6%)*
Missing	*2 (2.9%)*
Grade	
I	*11 (15.9%)*
II	*27 (39.1%)*
III	*12 (17.4%)*
Missing	*19 (27.5%)*
Relapse	
No	*44 (63.8%)*
Yes	*25 (36.2%)*
Type of surgery	
Palliative	*5 (7.2%)*
Whipple	*50 (72.5%)*
Other with curative intention	*14 (20.3%)*

### Immunohistochemistry

The paraffin-embedded pancreatic lesions were first sectioned on slides of 3 μm thickness and placed on SuperFrostPlus glass (Menzel–Glāser, Germany). The sections were de-paraffinized in xylene and rehydrated in a graded alcohol series and washed in 10 mM phosphate-buffered saline (PBS). To predigest the sections, they were placed in a microwave oven and boiled in 10 mM citric acid monohydrate (pH 6.0) for 12 minutes (8-OHdG) or in Tris-EDTA (pH 9.0) for 15 minutes (Keap1) and cooled for 30 minutes at room temperature. The sections were flooded in 1% hydrogen peroxide in methanol for 15 min (8-OHdG) or in 3% hydrogen peroxide in methanol for 10 min (Keap1) to consume the endogenous peroxide. The sections were incubated 1 hour at +37°C with mouse monoclonal 8-OHdG antibody (Japan Institute for the Control of Aging, Fukuroi, Japan) using 1:50 dilution. After washing with PBS the slides were incubated with biotinylated secondary antibody (Invitrogen Corporation, California, USA) for 20 minutes at room temperature and then, after washes, incubated for 20 minutes at room temperature in streptavidin-peroxidase (Invitrogen Corporation, California, USA). With Keap1, the slides were incubated with a 1:300 primary antibody dilution Keap1 E20 (Santa Cruz Biotechnology, Inc.) overnight at +4°C. The specificity of used antibody Keap1 E20 has been confirmed previously with western blotting [21,22]. Next morning the sections were incubated with Goat Probe (Biocare Medical, LLC, California, USA) for 15 minutes and after that the sections were incubated with Goat-on-Rodent HRP-Polymer (Biocare Medical, LLC, California, USA) for another 15 minutes. Dako Envision peroxidase detection system (Dako K5007) was used as a chromogen in both immunostainings and the sections were then counterstained with haematoxylin and finally mounted with Immu-Mount (Shandon, Pittsburgh, PA, USA).

Negative controls were produced by using the same procedure except that the primary antibodies (8-OHdG and Keap1) were replaced with PBS.

Immunohistochemical stainings were evaluated by experienced histopathogist (KMH). Both 8-OHdG and Keap1 immunostainings were divided to four class according to their intensity in malignant cells: − = no immunoreactivity observed; + = weak immunostaining; ++ = moderate immunostaining; +++ = strong immunostaining.

### Statistical analysis

IBM SPSS Statistics 21.0.0.0 for Windows was applied for statistical analysis. The reported p-values are from 2-sided chi-square tests, except for survival analysis. Survival was analyzed by using Kaplan-Meier curves with log-rank test and only pancreatic cancer-related death was used as an endpoint. Cox regression analysis was used in multivariate analysis. Relapse-free survival was calculated from the operation date to the confirmed date of relapse. T-class was divided in statistical analyses to either T1-T2 or T3-T4 and grade was divided into either grade I or grade II-III. Probability values below 0.05 were considered significant.

## Results

**Figure 1:**
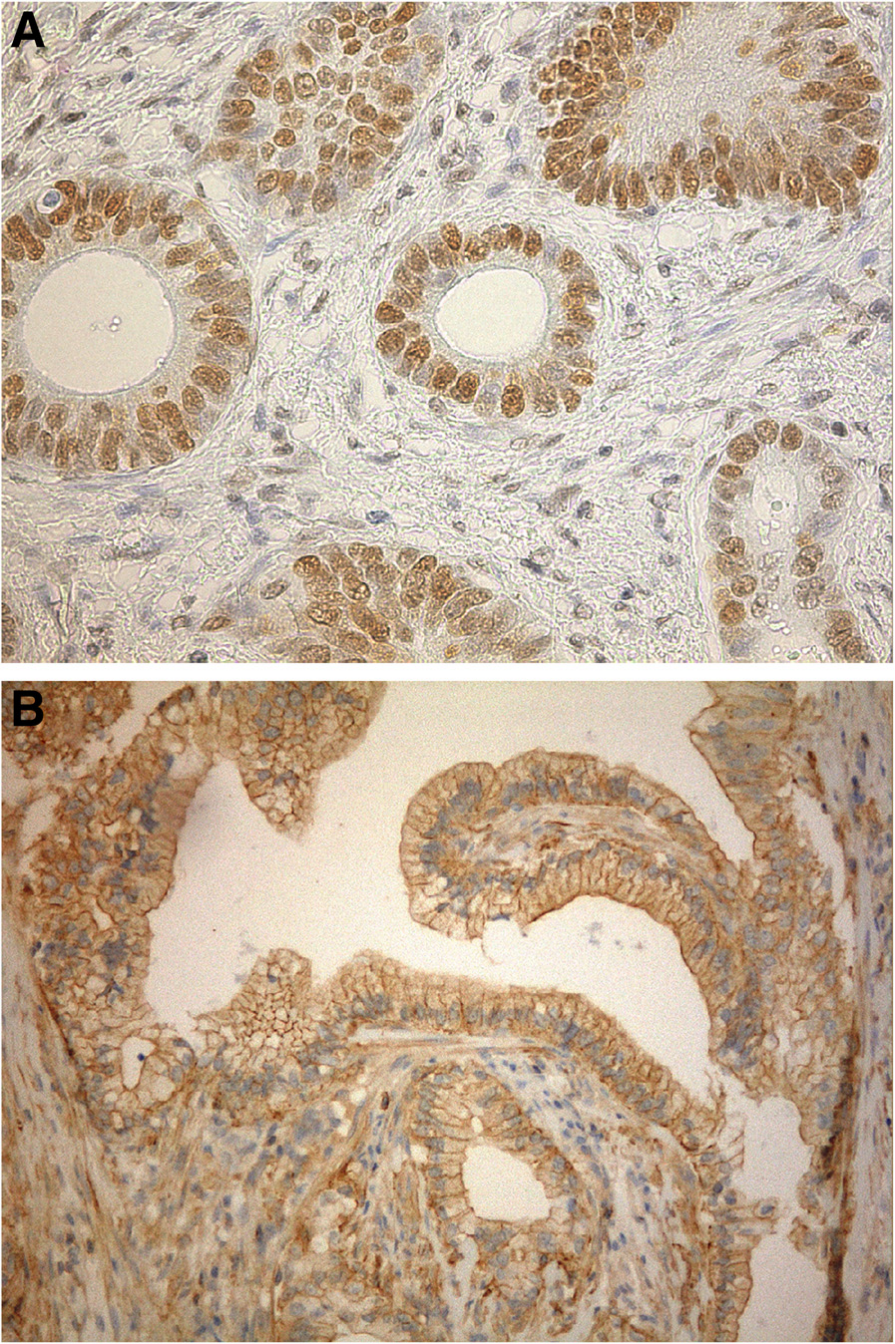
Strong nuclear positivity of 8-OHdG **(A)** and cytoplasmic and membrane bound Keap1 expression **(B)** in pancreatic ductal adenocarcinoma.

In malignant cells Keap1 immunostaining was found in cytoplasm and also on cell membranes. At places expression in membranes was even more prominent than in cytoplasm. Nuclear Keap1 immunoreactivity was not observed. 20 samples (29.0%) were totally negative for cytoplasmic Keap1, 11 (15.9%) were graded as 1+, 21 (30.4%) as 2+ and 12 (17.4%) as 3+. On cell membranes Keap1 was negative in 34 (49.3%) cases and 2+ in 30 (43.5%) cases, respectively. Keap1 expression was recorded in most cases in benign exocrine pancreas and in the islets of Langerhans. It was also located to smooth muscle tissue and to endothelium. Both cytoplasmic and membrane-associated reactivity were present also in benign tissues. In addition, neutrophils had notable cytoplasmic Keap1 expression. 8-OHdG expression was observed both in nuclei and cytoplasm of cancer cells, although nuclear staining was clearly more prevalent (Figure 1). 43 (62.3%) samples did not show any 8-OHdG expression in nuclei, 22 (31.9%) were graded as 2+ and 4 samples (5.8%) as 3+. In cytoplasm 8-OHdG was absent in 53 cases (76.8%), staining corresponding 1+ was recorded in one patient (1.4%), 2+ in 11 patients (15.9%) and 3+ in one patient (1.4%). Five Keap1 and three 8-OHdG immunostainings were missing due to exhaustion of blocks or because of other technical challenges.

Cytoplasmic Keap1 expression associated with the absence of lymph mode metastases (p = 0.0009) and distant metastases (p = 0.018) and non-significantly with high differentiation (grade I) (p = 0.075). Nuclear 8-OHdG immunostaining was overexpressed in patients with T1-T2 tumors (p = 0.016) and almost significantly in those without lymph node involvement (p = 0.051). Strong cytoplasmic 8-OHdG expression associated with grade I tumors (p = 0.023). Nuclear 8-OHdG and cytoplasmic Keap1 expressions associated with each other (p = 0.031).

**Figure 2:**
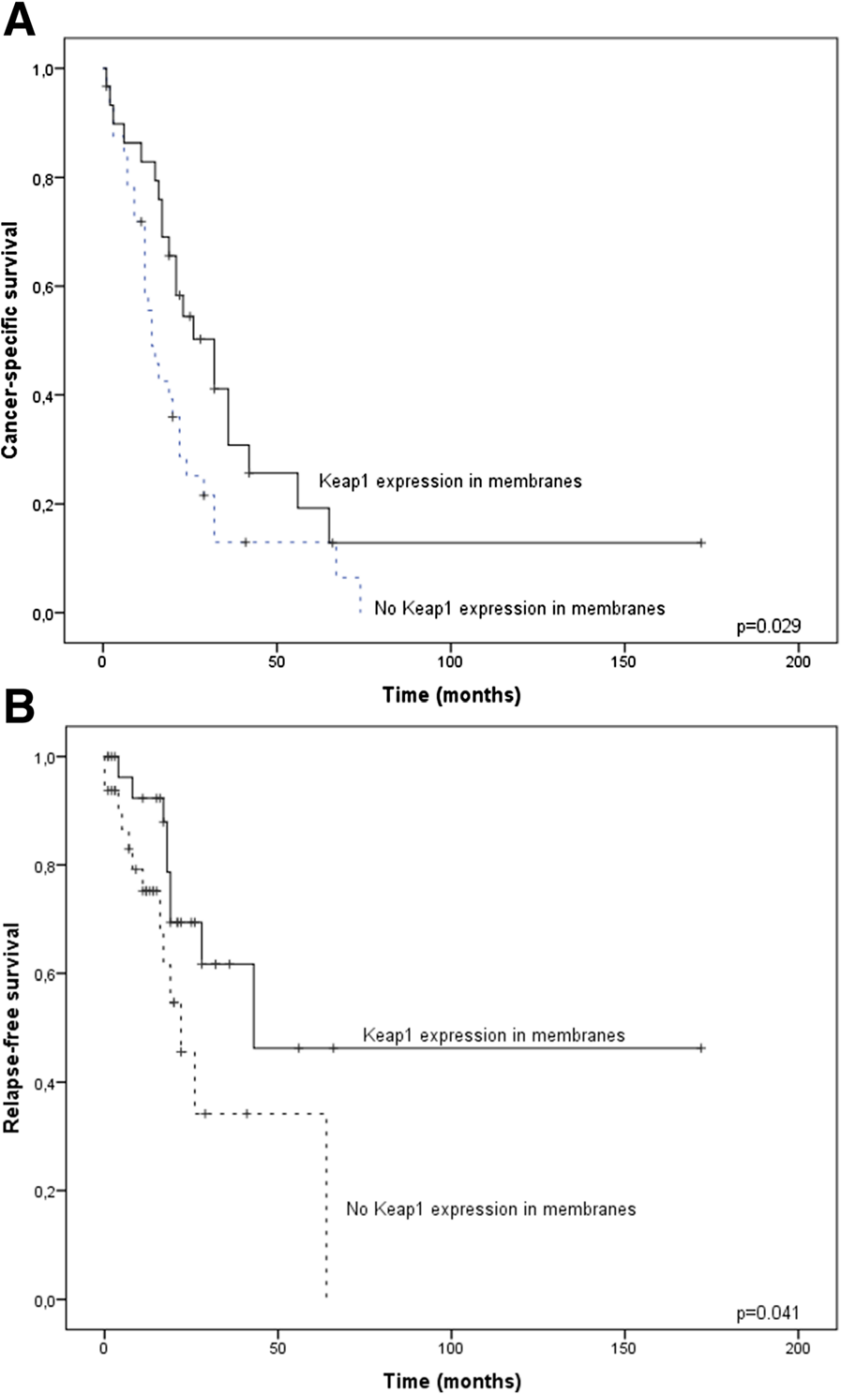
Kaplan-Meier curves showing pancreatic cancer-specific survival **(A)** and relapse-free survival **(B)** according to Keap1 expression in membranes.

Membranous Keap1 expression associated with better relapse-free survival (p = 0.041) and also with longer pancreatic cancer-specific survival (PCSS) (median survival 14 *vs*. 32 months; p = 0.029) (Figure 2). In Cox regression analysis negative membranous Keap1 expression was an independent prognostic factor of pancreatic cancer-related death (hazard ratio 2.66, 95% confidence interval 1.23-5.75) when grade, tumor size and lymph node involvement were taken into the model.

## Discussion

The present results show that expression of Nrf2’s main regulator, Keap1, is highly significant prognostic factor in pancreatic adenocarcinomas in terms of RFS and PCSS. These data give biological support to our recent study, which demonstrated the association of Nrf2 expression and poor survival [20].

Continuous activation of proto-oncogene Nrf2 and overexpression of Nrf2-targeted genes such as heme oxygenase-1 (HO-1), glutathione S-transferases and thioredoxin reductase 1 are observed in several solid carcinomas [23]. The alterations in the repressor of Nrf2, Keap1 may activate Nrf2-regulated antioxidant response pathway at least 1) by achievement of somatic Keap1 mutations to disturb Nrf2/Keap1 interaction 2) by epigenetic silencing via methylation of Keap1 resulting in nuclear accumulation of Nrf2 3) by cysteine residual modifications of Keap1 [15,18,23,24]. There are no recognized germ-line mutations of Keap1, but somatic mutations have been reported in various carcinomas [25]. In pancreatic carcinomas only synonymous mutations of Keap1 have been reported [26] but interestingly in the context of pancreatic cancer biology, Keap1 mutations in other cancers seem to be more considerably prevalent in adenocarcinomas and in patients with smoking history [25].

According to our knowledge, there is only one previous study evaluating Keap1 expression in pancreatic cancer in humans [26]. The authors reported no associations between Keap1 and clinicopathological parameters, however, material was smaller than ours and no survival analysis was performed. In the present study cytoplasmic Keap1 expression was tightly connected to reduced metastatic tendency, both to lymph nodes and to distant sites. Cytoplasmic Keap1 likely reflects Nrf2-bound complexes, which results in Nrf2’s ubiquitination and inhibition of its function and subsequently ARE and Nrf2 target genes are inactivated. These genes include HO-1, which play a central role in pancreatic cancer metastasis formation by inducing angiogenesis in co-operation with PTEN, which predicts worse survival in several studies [25,27]. The function of membrane-associated Keap1 is not as obvious, but previous immunolocalization study found Keap1 also as a component of cell-cell junctions and also in focal adhesions and the authors suggested that Keap1 may also have actin-binding properties [28].

Despite of protection against metastatic potential, cytoplasmic Keap1 expression did not have impact to survival curves. Instead, Keap1 expression associated in cell membranes had a significant protective effect on survival in multivariate analysis, with median pancreatic cancer-specific survival more than doubled. The magnitude of Keap1’s protective value may therefore be even more prominent than previously reported Nrf2 expression [20]. There were no long-time survivors in patients with negative membranous Keap1 immunostaining, whereas in those with Keap1 expression there was a plateau in survival curve during five follow-up years. Even more strikingly, in the group having membranous Keap1 expression, 46.3% of patients did not develop a relapse, which can be considered as a substantial proportion among these patients. Rare previous data from other carcinomas shows that in squamous non-small cell lung carcinoma Keap1 expression likewise associated with better survival with HR of 2.09 [29]. In contrast, we and others have previously shown that there is aberrant Keap1 methylation in triple-negative breast cancer (TNBC) and Keap1 overexpression is associated with poor prognosis in TNBC [30,31]. In breast cancer paradoxical connection between Keap1 and worse survival may derive from the sensitive induction of Keap1 in stressed tumors, rather than carcinogenesis promoting features of Keap1 itself as discussed previously [31].

Association between nuclear 8-OHdG and cytoplasmic Keap1 expressions probably derives from decreased nuclear DNA damage after Keap1 downregulation and resulting activation of Nrf2’s target genes in ARE. Former studies using Comet assay or HPLC-EC have suggested higher levels of oxidative DNA damage in pancreatic tumors than in benign pancreatic tissue [32], but studies correlating 8-OHdG and clinicopathological parameters or survival are lacking. We found no association between 8-OHdG expression and either RFS or PCSS, but the presence of nuclear 8-OHdG was connected to smaller primary tumor size and the absence of lymph node metastases. Cytoplasmic 8-OHdG also correlated with better differentiation. In nuclei 8-OHdG is considered as damage marker after ^•^OH attacks to guanosine and based on our recent data from transmission electron microscope studies on Hodgkin lymphomas, cytoplasmic 8-OHdG expression is likely to reflect mitochondrial DNA adducts and damage in mRNA, tRNA, or rRNA [33]. 8-OHdG is one of the most applied markers of oxidative stress and its immunohistochemical expression has been connected to poor survival in melanoma, colorectal cancer, diffuse large B-cell lymphoma and in ovarian carcinoma [34-39]. Nevertheless, excessive oxidative stress may lead to growth restriction or ultimately to apoptosis [11]. This has been suggested as a hypothesis why 8-OHdG expression is considerably attenuated in invasive breast carcinomas compared with non-invasive breast lesions and why high 8-OHdG levels have been constantly associated with better prognosis in breast carcinomas [30,38,39]. Furthermore, 8-OHdG expression is dependent from DNA repair enzymes, especially from the function of human 8-oxoguanine glycosylase (hOGG1). Association between more aggressive pancreatic carcinoma characteristics and lower 8-OHdG expression may therefore result from the induction of DNA repair enzymes under oxidative stress, although this hypothesis remains to be confirmed.

## Conclusions

In conclusion, this study adds data to the growing knowledge that highlights the importance of Keap1/Nrf2 pathway in human carcinomas. Although we had at least sufficient follow-up and carefully collected clinicopathogical data, sample size should be more considerable in future studies validating the current results. We also recommend the possible association between Keap1/Nrf2-axis and chemoresistance to be assessed in prospective studies.
